# Far beyond Phagocytosis: Phagocyte-Derived Extracellular Traps Act Efficiently against Protozoan Parasites* In Vitro* and* In Vivo*

**DOI:** 10.1155/2016/5898074

**Published:** 2016-06-30

**Authors:** Liliana M. R. Silva, Tamara Muñoz-Caro, Rafael A. Burgos, Maria A. Hidalgo, Anja Taubert, Carlos Hermosilla

**Affiliations:** ^1^Institute of Parasitology, Justus Liebig University Giessen, 35392 Giessen, Germany; ^2^Laboratory of Molecular Pharmacology, Institute of Pharmacology and Morphophysiology, Faculty of Veterinary Sciences, University Austral of Chile, Valdivia, Chile

## Abstract

Professional mononuclear phagocytes such as polymorphonuclear neutrophils (PMN), monocytes, and macrophages are considered as the first line of defence against invasive pathogens. The formation of extracellular traps (ETs) by activated mononuclear phagocytes is meanwhile well accepted as an effector mechanism of the early host innate immune response acting against microbial infections. Recent investigations showed evidence that ETosis is a widely spread effector mechanism in vertebrates and invertebrates being utilized to entrap and kill bacteria, fungi, viruses, and protozoan parasites. ETs are released in response to intact protozoan parasites or to parasite-specific antigens in a controlled cell death process. Released ETs consist of nuclear DNA as backbone adorned with histones, antimicrobial peptides, and phagocyte-specific granular enzymes thereby producing a sticky extracellular matrix capable of entrapping and killing pathogens. This review summarizes recent data on protozoa-induced ETosis. Special attention will be given to molecular mechanisms of protozoa-induced ETosis and on its consequences for the parasites successful reproduction and life cycle accomplishment.

## 1. Introduction

Professional mononuclear phagocytes, such as polymorphonuclear neutrophils (PMN), monocytes, and macrophages, are considered as the first line of defence of the early host innate immune response [1, 2]. Their main function has been classically understood to kill invasive pathogens by a variety of potent intracellular microbicidal effector mechanisms [3–7]. After the first contact with pathogens, mononuclear phagocytes engulf and internalize them into their phagosomes. By the fusion with intracellular granules and the formation of phagolysosomes the pathogens may be killed intracellularly by a combination of non-oxidative and oxidative mechanisms [1, 8]. Actions of potent antimicrobial peptides, such as defensins, cathelicidins, cathepsins, pentraxin, and lactoferrin, are parts of non-oxidative killing mechanisms, while oxidative killing relies exclusively on the production of antimicrobial reactive oxygen species (ROS) via the NADPH oxidase (NOX) complex [5]. Within blood circulating phagocytes, PMN are by far the most abundant cell population representing 50–80% of the total white blood cells in different vertebrates [5]. Moreover, after being released from the bone marrow into the blood circulation, PMN are highly mobile and short-lived phagocytes, being densely packed with secretory granules [4, 8]. PMN granules are categorized into three different types based on their contents: primary (azurophilic), secondary (specific), and tertiary (gelatinase) granules. The types of granules to be found in circulating PMN depend on their maturation stage. Thus, PMN maturation starts with the formation of primary granules, followed by secondary and tertiary granules [4, 9, 10]. The content of primary granules includes myeloperoxidase (MPO), neutrophil elastase (NE), cathepsin G, proteinase 3, defensins, and lysozyme; secondary granules contain collagenase, gelatinase, cystatin, lysozyme, and lactoferrin; and tertiary granules comprise gelatinase, lysozyme, and arginase amongst others [10]. Following granule maturation, PMN will possess all three types of granules displaying full killing capacity not only in the blood but also in tissues/organs and gut lumen [10].

In addition, PMN act against pathogens by actively participating in complex inflammatory networks such as the release of a broad panel of proinflammatory chemokines, cytokines, and survival- and growth-factors which trigger both downstream proinflammatory effects and the transition into adaptive immune reactions. As such, several proinflammatory cytokines/chemokines were found enhanced in activated PMN in response to apicomplexan parasites, such as TNF-*α*, IL-1*β*, CC, and CXC chemokines (e.g., IL-8, IP-10, GRO-*α*, RANTES, and MIP-1*α*) [11–15]. Several of PMN-derived immunomodulatory molecules can augment the production of various chemokines and cytokines to further regulate phagocyte functions [16, 17]. More importantly, by this means activated PMN recruit not only other innate immune cells but also T cells to the site of infection [18–20] or even induce sterile inflammation [21, 22].

## 2. Neutrophil Extracellular Traps (NETs) and Phagocyte-Derived Extracellular Traps (ETs)

Beginning with the landmark study of Brinkmann et al. [31], the paradigm of how PMN fight and kill pathogenic bacteria has profoundly been changed. The discovery of DNA-based antimicrobial neutrophil extracellular traps (NETs) changed the current knowledge on innate immune reactions not only on the level of the pathogen killing but also on the pathophysiology of metabolic, autoimmune, reproductive, and inflammatory diseases, as well as cancer progression [32–37]. NETs are released by activated PMN by a novel cell death process, called NETosis [38], which can be stimulated by a variety of molecules and invasive pathogens. Microorganisms such as bacteria [31, 39–41], fungi [42–44], viruses [45–49], and parasites [50–55] were identified as NET inducers. Also different molecules or cellular structures such as GM-CSF/complement factor 5a [56, 57], activated platelets [40, 58], PMA and zymosan [24, 26, 31, 59], singlet oxygen [60], LPS [31, 61], and Fc receptor [42] trigger NETosis. In addition, IL-8 as well-known chemoattractant for PMN was demonstrated as NET inducer [31, 62]. Efficient NETosis requires mature PMN and in most cases NOX, MPO, NE, and peptidylarginine deiminase type IV (PAD4) activities [14, 24, 59, 63–65]. Furthermore, the process of NETosis obviously requires intracellularly signalling pathways of which Raf-MEK-ERK kinases as well as p38 MAPK are being the most frequently reported to be involved in this process [14, 23, 33, 66–69]. In addition, calcium release is needed for optimal NET formation in different vertebrate species [14, 23, 70–72]. Upon stimulation of PMN, the nuclear envelope disintegrates permitting the mixture of chromatin with granular proteins/peptides [38]. NE and MPO degrade histones (H1, H2A/H2B, H3, and H4) and promote chromatin decondensation [65], mediated by PAD4 via hypercitrullinating of specific histones to allow electrostatic coiling of the chromatin [64, 73, 74]. The total of the DNA complexes being decorated with granular proteins/peptides and specific histones (H1, H2A/H2B, H3, and H4) are finally extruded as NETs to the extracellular environment by dying PMN.

NET formation is primarily a NOX-dependent mechanism [14, 24, 59, 75, 76]. However, NOX-independent NETosis was also reported [29, 60, 67, 68, 77]. This mode of NETosis is accompanied by a substantially lower level of ERK activation and rather moderate level of Akt activation, whereas activation of p38 is similar in both kinds of NET formation [67, 68]. As an example, singlet oxygen can stimulate NETosis in a NOX-independent manner [60]. Irrespectively of NOX-dependency, pathogens may either be immobilised within sticky DNA fibres [55, 78, 79] or be killed via the local high concentration of effector molecules [31, 42, 51, 53].

Meanwhile, other types of leukocytes of the innate immune system, such as macrophages [80–83], monocytes [26, 28], mast cells [84, 85], eosinophils [55, 86, 87], and also basophils [88], have been reported to release NET-like structures which are now collectively entitled as extracellular traps (ETs).

Interestingly, Malawista et al. [89] described already many years ago that enucleated PMN may remain vital and are even capable of killing invasive microbes. More recent studies corroborated these findings proving that leukocytes do not necessarily die after ET extrusion [56, 68, 86]. In this context, Yousefi et al. [56, 86] demonstrated that eosinophils and certain PMN subpopulations release ETs of mitochondrial origin without dying. Furthermore, Yipp et al. [90] verified that PMN which had released NETs were still viable and retained their capability to engulf bacteria via phagocytosis. The precise mechanism of NOX-independent NET formation is not clear yet. However, it appears to be nonlethal for PMN and faster than NOX-dependent NET formation and to rely on a vesicular-based pathway releasing nuclear DNA [33, 68].

Additionally, different molecular pathways will lead in a stimulus-dependent manner to the extrusion of different types of ETs* in vitro* and* in vivo*. Different morphological forms of ETs were for the first time described in the human gout disease* in vivo* proving that monosodium urate crystals (MSU) induced aggregated (*agg*ETs), spread (*spr*ETs), and diffused (*diff*ETs) ET formation [91]. Consistently, also parasite-mediated ETosis resulted in different types of ETs. As such, the parasitic nematode* Haemonchus contortus* larvae triggered in ruminant PMN and eosinophils* agg*ETs,* spre*ETs, and* diff*ETs [55].

## 3. Apicomplexan Protozoa-Induced Formation of NETs and ETs

### 3.1. Plasmodiidae

While most NET- and ET-related studies focused on bacterial, viral, and fungal pathogens, little attention was paid to protozoan parasites. As such, the first ever published study on parasite-triggered NETosis was published in 2008 by Baker et al. [50] 4 years after the discovery of this novel effector mechanism [31] and reported on* Plasmodium falciparum*-triggered NET formation.

*Plasmodium* spp. parasites are mosquito-borne pathogens that cause malaria, a serious public health disease worldwide in the tropic and subtropics. Globally, an estimated 3.3 billion people are at risk of being infected with malaria of whom approximately 1.2 billion are at high risk (>1 in 1000 chance) of developing malarial disease [92]. The first report on* P. falciparum*-induced NETs referred to* P. falciparum-*infected children and demonstrated* in vivo* NET-entrapped trophozoite-infected erythrocytes in blood samples [50]. Moreover, Baker and colleagues [50] provided first evidence on the involvement of parasite-triggered NETs in the pathogenesis of malaria since the high levels of anti-dsDNA antibodies were above the predictive levels for autoimmunity. Interestingly, a recent study also indicates the capacity of* P. falciparum* to inhibit NET formation [93] which may be of relevance in immunopathogenesis. Thus, a mosquito-derived salivary protease inhibitor (agaphelin) induced by* P. falciparum* infection inhibited vertebrate elastase and NET formation [93]. Whether this represents a true anti-NET mechanism remains to be elucidated.

### 3.2. Eimeriidae

Parasites of the genus* Eimeria* are worldwide of high veterinary and economic importance in livestock, especially in chicken [94], cattle and small ruminants [95–100]. Coccidiosis is a disease with high morbidity in animals of all ages, nonetheless inducing pathogenicity especially in young animals [101] and occasionally causing death of heavily infected animals [99, 102, 103].

**Figure 1:**
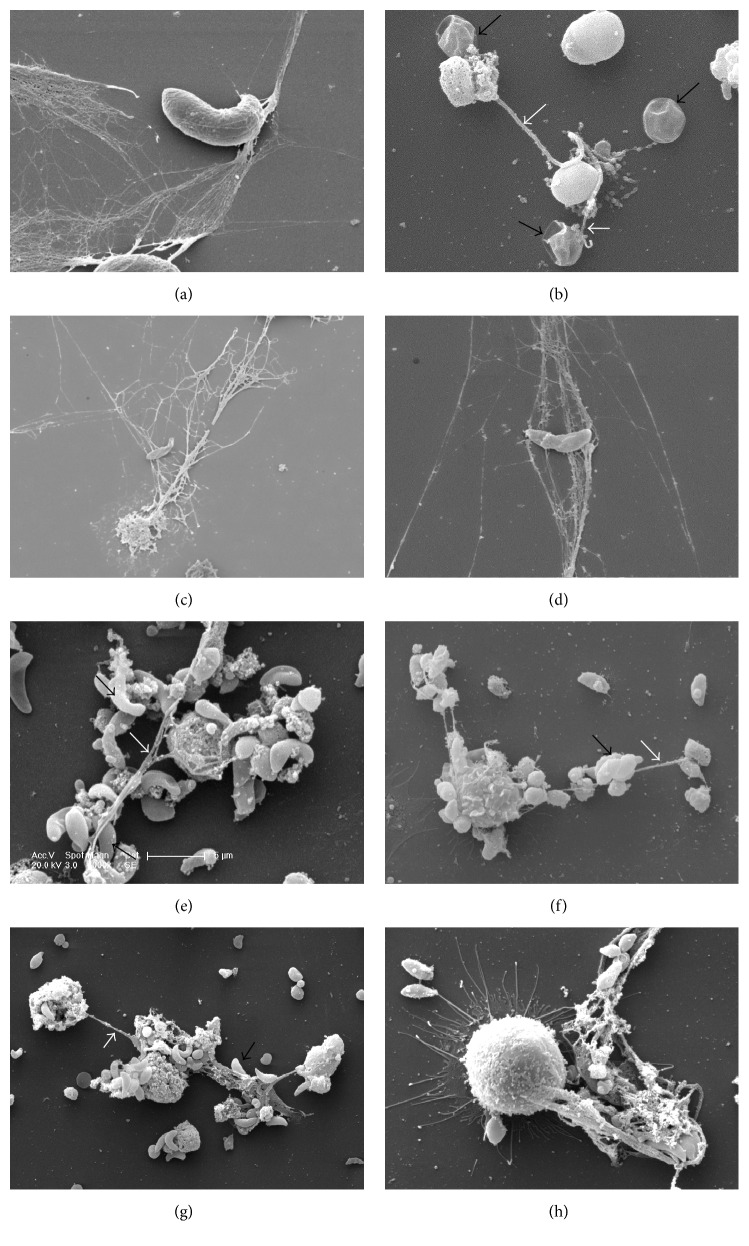
ETosis is not a parasite- nor a stage-specific cell death process (SEM analysis). (a)* Eimeria bovis* sporozoite-triggered bovine NETosis; (b)* Cryptosporidium parvum* oocysts (back arrows) induced NETosis (white arrows); (c)* Toxoplasma gondii* tachyzoites entrapped by a delicate DNA fibre derived from bovine PMN; (d)* Toxoplasma gondii* tachyzoite completely entrapped in filigree NET structures; (e) PMN-derived NETs (white arrow) after* Besnoitia besnoiti* tachyzoites encounter (black arrows); (f)* Neospora caninum* tachyzoites (black arrow) trapped in bovine NETs (white arrow); (g) monocyte-derived extracellular traps (METs) forming spread (white arrow) ETs entrapping* Besnoitia besnoiti* tachyzoites (black arrow); (h)* Besnoitia besnoiti* derived thick and thin METs.

**Figure 2:**
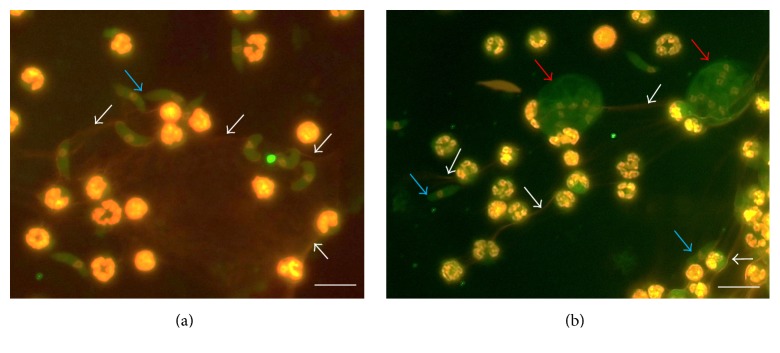
Colocalization of caprine NET-derived DNA and MPO. (a) Cocultures of caprine PMN and* Eimeria arloingi* sporozoites (blue arrows) were fixed, permeabilized, stained for DNA using Sytox Orange, and probed for MPO using anti-MPO along with adequate conjugate systems (white arrows); (b) cocultures of caprine PMN exposed to sporulated* E. arloingi*-oocysts (red arrows) and sporozoites (blue arrows). Filigree spread NET structures are indicated by white arrows. Bar scale = 20 *μ*m.

Several studies showed that PMN infiltrate intestinal mucosa in response to* Eimeria* infections and are occasionally found in close contact to the parasitic stages* in vivo* [102, 104–107]. PMN have also been shown to directly interact with* E. bovis* stages and antigens* in vitro*, resulting in release of proinflammatory cytokines, chemokines, and iNOS [13]. Additionally, their phagocytic and oxidative burst activities were enhanced in response to* Eimeria* stages* in vitro* and* in vivo* [13]. First indications on* Eimeria* spp. as potent NET inducers came from Behrendt and colleagues who reported on sporozoites to be entangled by an extracellular network of delicate DNA fibres being extruded from PMN* in vitro* (Figure 1(a)) [52]. Using extracellular DNA measurements and DNase treatments the authors presented strong indications that these structures were NETs. Other studies confirmed typical characteristics of NETs, such as the colocalization of NE, MPO, and histones in the DNA backbone of* Eimeria*-induced NET-like structures [23]. Meanwhile, also other pathogenic ruminant* Eimeria* species were shown to induce NETosis, such as* E. arloingi* (Figures 2(a) and 2(b)) [24, 27] and* E. ninakohlyakimovae* (Pérez, personal communication). Importantly, Muñoz-Caro and colleagues proved NETs also to occur* in vivo* in* Eimeria*-infected gut mucosa [27]. The current data suggest that* Eimeria*-induced NETosis is a species- and stage-independent mechanism, since it was induced by sporozoites, merozoites I, or oocysts of different* Eimeria* species [23, 24]. Given that PMN were described to act even in the intestinal lumen via different effector mechanisms [27, 108, 109], it appears likely that interactions of luminal PMN with ingested* Eimeria* oocysts or newly excysted sporozoites may occur [6, 23, 24]. In particular, NET-related reactions against oocysts would have a high impact on the ongoing infection since they may hamper proper excystation of infective stages (sporozoites) and, in consequence, dampen the degree of infection at the earliest possible time point in the host. Since* E. arloingi* sporozoites must egress from the oocyst circumplasm through the micropyle [24], NETs covering this area of the oocyst will have a detrimental effect on proper excystation [6, 24]. The same explanation seems feasible for* E. bovis* and* E. ninakohlyakimovae*, regardless of the fact that excystation occurs by rupture of the oocyst walls prior to sporozoites egress from sporocysts. Although all* Eimeria* species tested so far equally induced NETs, significant differences in entrapment effectivity were reported amongst different host species, parasite species, and stages. Thus, caprine NETs immobilised a high proportion of* E. arloingi* sporozoites (72%) [24], whilst in the bovine system considerably less parasite stages (*E. bovis* sporozoites: 43%,* B. besnoiti* tachyzoites: 34%) were found entrapped in NET structures [23, 59]. So far, it remains to be elucidated whether the varying effectivity of NETs is based on the PMN origin (goats are generally considered as strong immune responders) or on the parasite species.

The molecular basis of* Eimeria*-induced NETosis is not entirely understood, so far. Enzyme activity measurements and inhibition studies revealed a key role of NOX, NE, and MPO in* Eimeria*-triggered NET formation (see Table 1) which is in agreement to bacterial, fungal, and parasitic pathogens [14, 25, 59, 65, 75, 110]. Referring to signal cascades, analyses on the grade of phosphorylation revealed a key role of ERK1/2 and p38 MAPK in sporozoite-exposed bovine PMN. Since respective inhibitor experiments led to decreased parasite-mediated NET formation, Muñoz-Caro et al. [23] proved the relevance of this signalling pathway in sporozoite-triggered NETosis. This finding is in agreement with data on* T. gondii*-mediated NET formation [25]. Referring to Ca^2+^ influx, further inhibition experiments proved* E. bovis*-mediated NETosis as dependent on intracellular Ca^2+^ mobilization, since 2-ABP (inhibitor of store-operated Ca^2+^ entry) [23] and BAPTA-AM (binding intracellular Ca^2+^; Muñoz-Caro, unpublished data) but not EGTA (inhibitor of Ca^2+^ influx from the extracellular compartment; Muñoz-Caro, unpublished data) significantly blocked parasite-triggered NETosis. So far, little is known on PMN-derived receptors mediating parasite-triggered NETosis. Muñoz-Caro et al. [23] reported on enhanced CD11b surface expression on PMN following* E. bovis* sporozoite exposure. By antibody-mediated CD11b blockage leading to a significant reduction of parasite-triggered NETosis, the authors proved the relevance of this receptor in the NET formation process.

Bacteria and fungi NETosis was reported as a lethal effector mechanism [31, 42]. However, killing effects of NETs were not observed in the case of* Eimeria* spp. so far. Given that* Eimeria* spp. are obligate intracellular parasites, the main function of NETs rather seems to be the extracellular immobilisation of infective stages hampering them from host cell invasion. Accordingly, reduced host cell infections rates were reported for* E. bovis* and* E. arloingi* sporozoites when previously exposed to PMN [23, 24]. The same feature was reported for monocyte-preexposed* E. bovis* sporozoites indicating that this leukocyte cell type also casts ETs in response to this parasite stage and that ETosis had an impact on parasite invasion [28]. Besides* E. bovis* [59],* E. arloingi* (Silva, unpublished data), and* E. ninakohlyakimovae* (Pérez et al., submitted manuscript) were also shown to induce monocytes-derived ETs. Furthermore,* E. ninakohlyakimovae*-induced monocytes-ETosis showed a rapid induction of ETs release upon viable sporozoites, sporocysts, and oocysts encounters, corroborating a stage-independent process in monocyte-derived ETosis. In addition, it was found that caprine monocyte-derived-ETosis is NOX-dependent. With the upregulation of the genes transcription encoding for IL-12 and TNF-*α*, relevant immunoregulatory cytokines with transition properties into the adaptive immunity [111] were also demonstrated in* E. ninakohlyakimovae-*exposed caprine monocytes (Pérez et al, submitted manuscript).

Since the reduction in infection rates early after infection automatically results in decreased proliferation of the parasite, this indirect ET-mediated effect should have a beneficial impact on the outcome of the disease. Despite advantageous properties of ETs, their ineffective clearance and/or poor regulation might also bear adverse pathological implications, leading to tissue damage in addition to enhanced local proinflammatory reactions [112, 113].

### 3.3. Sarcocystidae

Toxoplasmosis is caused by the facultative heteroxenous apicomplexan polyxenous protozoan* T. gondii* representing one of the most common parasitic zoonoses worldwide [114].* Toxoplasma gondii* is well known to affect almost all warm-blooded mammals including a wide range of domestic animals, wild mammals, marine mammals, marsupials, and humans [115, 116]. In response to* T. gondii* infections, PMN are promptly recruited to the site of infection producing a variety of proinflammatory cytokines and chemokines [11, 117]. In addition, PMN are capable of killing* T. gondii* tachyzoites via phagocytosis [118, 119]. Besides this effector mechanism, human, murine, bovine, and harbour seal (*Phoca vitulina*) PMN additionally perform NETosis in reaction to* T. gondii* tachyzoites (Figures 1(c) and 1(d)) [25, 26]. Abi Abdallah et al. [25] showed that NETosis was triggered by tachyzoites in a parasite strain-independent fashion as an invasion/phagocytosis-independent process. Interestingly, in the murine toxoplasmosis model, tachyzoites-induced NETs were not the result of a random cell lysis, but of a controlled DNA release process since lysozyme was still present in PMN after performing NETosis [25, 120]. In contrast to* Eimeria* spp.,* T. gondii-*triggered NETosis had modest toxoplasmacidal effects by killing up to 25% of the parasites [25]. Considering the obligate intracellular life style of* T. gondii* and its enormous proliferative capacity in mammalian host cells, parasite entrapment via NETs might be of particular importance* in vivo* based on its interference with host cell invasion. Consistently, harbour seal PMN-promoted NETs significantly hampered host cell invasion of* T. gondii* tachyzoites* in vitro* [26].* In vivo* evidence of* T. gondii*-induced NETosis was reported in a murine pulmonary infection model, revealing an increase of dsDNA contents in the bronchoalveolar lavage fluids of* T. gondii*-infected mice [25]. As equally reported for several other coccidian parasites [14, 23],* T. gondii*-induced NETs were also proven to be NOX-, NE-, MPO-, and Ca^2+^- (SOCE) dependent and to be mediated by an ERK 1/2-related signalling pathway in PMN (see Table 1) [25, 26]. Additionally, in earlier studies, not only the pivotal role of PMN but also the important role of monocytes in toxoplasmosis was clearly demonstrated [121–123]; however, their capacity to also induce ETs in response to tachyzoite stages was just recently demonstrated [26]. Exposure of harbour seal-derived monocytes to viable* T. gondii* tachyzoites resulted in a significant induction of monocyte-ETs and tachyzoites were firmly entrapped and immobilised within harbour seal monocyte-ET structures, hampering parasite replication [26].

Bovine besnoitiosis caused by* Besnoitia besnoiti* is an endemic disease in Africa and Asia [124–126] and considered as emergent in Europe [127]. During the acute phase of cattle besnoitiosis,* B. besnoiti* tachyzoites mainly replicate in host endothelial cells of different organs [28, 128] and, upon release, may be exposed to circulating leukocytes.* Besnoitia besnoiti* tachyzoites were recently reported as effective inducers of PMN- and monocyte-derived ETs (Figures 1(e), 1(g), and 1(h)) [28, 59]. In the latter case, ETosis was further reported to occur as an invasion- and phagocytosis-independent process [28]. A high proportion of PMN was found to be involved in NETosis, since up to 76% of encountered PMN were found to participate in NETosis leading to the immobilisation of approximately one-third of the parasites [59].* Besnoitia besnoiti*-triggered NETosis furthermore proved as vitality-independent process that was even induced by soluble parasite molecules (homogenates), though at lower levels [59]. Regarding PMN-derived effector molecules, NOX, NE, and MPO proved as essential for efficient* B. besnoiti*-triggered NETosis [59]. Thus, respective enzyme activities were encountered in tachyzoite-exposed PMN and chemical blockage of these enzymes via inhibitors blocked parasite-triggered NETosis [28, 59]. In contrast to tachyzoites of* T. gondii*, entrapped* B. besnoiti* tachyzoites were neither killed by NETs nor ETs since their host cell infectivity was entirely restored upon DNase I treatments [28, 59].

Given that* B. besnoiti* tachyzoites mainly proliferate within endothelial cells during the acute phase, these parasitic stages are released via cell lysis in close proximity to endothelium and are exposed to blood contents, such as leukocytes. Several reports have shown that NETs themselves interact with endothelium and may cause endothelial damage or dysfunction [129–131]. Since activated endothelial cells may produce a broad panel of immunomodulatory molecules with IL-8 or P-selectin having been identified as potent NET inducers [129, 132], interactions between infected endothelial cells,* B. besnoiti* tachyzoites, and NETs are quite likely. Accordingly, Maksimov et al. [15] recently reported on infection-induced upregulation of endothelial-derived IL-8 and P-selectin gene transcription and furthermore presented indications on NET formation occurring adjacent to infected endothelium after PMN adhesion assays being performed under physiological flow conditions as the ones present in small vessels.

Recent NET-related investigations on the closely related cyst-forming apicomplexan protozoa* Neospora caninum* have shown that bovine PMN exposed to viable tachyzoites also result in strong NETosis (Figure 1(f)). With regard to molecular mechanisms,* N. caninum*-triggered NETosis seems to be P2Y2-, NOX-, SOCE-, MPO-, NE-, ERK1/2-, p38 MAPK-, and PAD4-dependent (Villagra-Blanco et al., submitted manuscript).

### 3.4. Cyptosporiidae

*Cryptosporidium parvum* is an euryxenous apicomplexan parasite with worldwide distribution and high zoonotic potential, mainly affecting young children, immunocompromised humans, and neonatal livestock [133]. Typically, cryptosporidiosis is a water- and food-borne enteric disease that causes diarrhoea, dehydration, weight losses, and abdominal pain and leads to significant economic losses in the livestock industry [133, 134]. After ingestion, sporozoites are released from oocysts into the intestinal lumen and infect small intestine epithelial cells [135]. Recent studies reported on a significant contribution of PMN and macrophages to inflammatory responses in cryptosporidiosis* in vivo* [136, 137]. Muñoz-Caro and colleagues reported on NETs being cast by both bovine and human PMN in response to* C. parvum* stages [14]. Parasite-triggered NETosis proved stage-independent since it was induced by both sporozoites and oocysts (Figure 1(b)). Especially in the latter case parasite stages were occasionally entirely covered with NET structures thereby most probably hampering proper sporozoite excystation [14]. Given that PMN were shown as active even within the intestinal lumen [108, 109, 138, 139], these reactions should have a significant impact on ongoing* in vivo* infection.* In vitro* infection experiments additionally showed the negative impact of NETs on host cell invasion since infection rates were significantly reduced when using PMN-preexposed* C. parvum* stages [14]. The fact that these reactions were entirely reversible via DNase I treatments rather argued against any cryptosporidicidal effects of NETs [14]. The colocalization of NE, histones, and MPO with DNA in parasite-mediated extracellular fibres proved classical characteristics of NETs and inhibitor experiments emphasized the key role of NE, NOX, and MPO in efficient NET formation [14]. In agreement with findings on* Eimeria*-induced NETosis, inhibition experiments revealed* C. parvum*-triggered NET formation as dependent on intracellular Ca^2+^ release and ERK 1/2 and p38 MAPK-mediated signalling pathways [14]. Interestingly,* C. parvum* sporozoite-exposed bovine PMN showed increased gene transcription of proinflammatory molecules, some of which were recently shown as potent NET inducers (e.g., IL-8 and TNF-*α*) [140, 141] and may have potentiated NET reactions.

## 4. Euglenozoan Protozoa-Induced NETs

### 4.1. Trypanosomatidae

Infections with* Leishmania* spp. represent a major health problem and according to the WHO [92] 10% of the human world population is at risk of infection, meaning that approximately 12 million people in 98 countries are infected, and 2 million new cases occur each year [142, 143]. Leishmaniasis is a vector-transmitted zoonosis caused by more than 25 different obligate intracellular protozoan* Leishmania* species [142–144]. Particularly PMN have been implicated in the immunopathogenesis of leishmaniasis [145–149] and recent studies examined the potential role of NETs during the early phase of the disease of different* Leishmania* species. Guimarães-Costa et al. [51] showed for the first time that promastigotes of* Leishmania amazonensis*,* L. major*, and* L. chagasi* were capable of triggering NET formation. Additionally,* Leishmania-*triggered NETosis seems not entirely stage-specific, since both promastigotes (*L. amazonensis*,* L. major*,* L. chagasi*,* L. donovani*,* L. mexicana*, and* L brasiliensis*) and amastigotes (*L. amazonensis*,* L. braziliensis*) promoted NET formation* in vitro* and* in vivo* [51, 147, 150–152]. More importantly, Guimarães-Costa et al. [51] provided first indications on possible parasite-specific ligands being responsible for* Leishmania-*mediated NETosis. Thus,* Leishmania-*derived lipophosphoglycans (LPG) were suggested as the main trigger of NET release since these molecules also induced NETs in a purified form. The former authors showed that NETs possessed detrimental effects on parasites as NET-entrapped* L. amazonensis* promastigotes exhibited decreased viability [51]. Authors also demonstrated that the extracellular DNA and histones found on NETs were involved in the parasite inactivation/killing process [51]. The leishmanicidal effects of histones were proven in promastigotes cocultures with purified H2A histones leading to the killing of parasites and by a significant reduction of leishmanicidal effects when cocultured in the presence of anti-histone antibodies. Additionally to H2A histone killing effects, Wang et al. [153] demonstrated that also the histone H2B could directly and efficiently kill promastigotes of* L. amazonensis*,* L. major*,* L. braziliensis,* and* L. mexicana*.

In case of* L. donovani*, Gabriel et al. [150] reported NETosis as a ROS-dependent process which was equally triggered in human and murine PMN (see Table 1). However,* Leishmania*-lipophosphoglycan- (LPG-) dependent NET induction reported by Guimarães-Costa et al. [51] was not observed with* L. donovani*. When using genetically modified* L. donovani* promastigotes Gabriel et al. [150] observed a lipophosphoglycan- and GP63-independent (promastigote surface metalloprotease) NETosis pathway. Nonetheless, in this infection system, LPG appeared to be involved in the resistance to NETs-mediated killing, since the wild type of* L. donovani* maintained its viability in the presence of NETs, whilst mutant parasites lacking LPG were efficiently killed by these extracellular structures [150].

A more recent study revealed that* Leishmania* parasites trigger not only the classical ROS-dependent NETosis as previously demonstrated but also a ROS-independent form, named as early/rapid vital NETosis [29]. During this early/rapid* Leishmania-*triggered NETosis, in which NET formation takes place after 5–15 min of activation without affecting PMN viability [29, 68], the parasites are also being efficiently entrapped.

Regarding NET-related evasion strategies of Trypanosomatidae parasites,* Leishmania* spp. seem capable of evading NET killing by firstly blocking the oxidative burst activity of PMN or even by resisting microbicidal activity of NETs [145, 150]. Moreover, Guimarães-Costa et al. [152] showed that* L. infantum* promastigotes express the enzyme 3′-nucleotidase/nuclease which was previously described to be involved in parasite nutrition and infection and was proven to be part of the ability of promastigotes to escape NET-mediated killing. A recent investigation has shown that a salivary component of the sand fly insect that transmits leishmaniasis may also play a role in the survival of* Leishmania* in the definitive hosts, by modulating their innate immune system. A molecule named Lundep from the salivary gland of* Lutzomyia longipalpis* was recently described as an endonuclease with NET-destroying properties in humans [145]. In the presence of Lundep, human NETs were disrupted, thus increasing* L. major* survival rates [145]. Furthermore, Chagas et al. [145] measured the NE release from NETs as an indicator of NET destruction, since NE is normally decorating NETs backbone structures and found at low concentrations in culture supernatants, as previously demonstrated [39]. Lundep was responsible for the significant increase of NE concentration in the supernatants when compared to negative controls [145]. In conclusion, these experiments showed degradation of DNA scaffold of NETs, destroying their functional integrity, and increasing promastigote survival and exacerbating* L. major* infection.

American trypanosomiasis or Chagas disease is caused by the protozoan parasite* Trypanosoma cruzi*. Approximately eight million people are affected by this tropical disease in the Americas and an average of 12,000 deaths per year is known to occur due to American trypanosomiasis [154]. It is well known that macrophages, eosinophils, monocytes, and PMN are implicated in the control of early infection [30, 155]. Recently, Sousa-Rocha et al. [30] demonstrated* in vitro* that* T. cruzi* is able to trigger NETs in a dose-, time-, and ROS-dependent manner. In agreement with reports on* Eimeria* spp. and* B. besnoiti *[23, 24, 59] but in contrast to observations on* T. gondii* and* Leishmania* spp. [25, 51], the viability of* T. cruzi* stages was not affected by NETs, but NETosis significantly impaired the parasite host cell infectivity. In fact, NETs components as NE may affect* T. cruzi* infectivity, since this enzyme appears to be involved in increased trypanocidal activity and in the reduction of trypomastigote release by prestimulated infected macrophages [30, 156]. Additionally, the authors showed via antibody-mediated blockage that* T. cruzi*-triggered NETosis is a TLR2- and TLR4-dependent process. Moreover, the study showed that not only viable* T. cruzi* trypomastigote forms but also soluble antigens and killed* T. cruzi* parasites induced NET release in human PMN.* In vivo* murine studies indicated the relevance of NETosis for the outcome of trypanosomiasis since significantly decreased parasites numbers were found in the blood system of those animals which had previously been infected with NETs-pretreated parasites [30].

## 5. Conclusions

During the last years a vast amount of data on protozoan-mediated ETosis was published strengthening the role of this effector mechanism in the defence of parasitic infections. Several* in vivo* data have now proven the existence and importance of this early host innate effector mechanism. However, there is still a total lack of information on parasite-derived ligands triggering ETosis. Taking into account that in most cases ET formation is considered as a species- and stage-independent process, rather ubiquitary occurring molecules may represent parasite-derived target molecules of ETs. Moreover, recent data revealed that other leukocytes such as monocytes, macrophages, basophils, mast cells, and eosinophils also perform ETosis upon pathogen encounter. However, respective data on parasite-induced mechanism are scarce. Furthermore, ET-related research mainly focused on the leukocytes aptitude to impact the parasites life cycle, but not on the propensity of parasitic stages to develop counter mechanisms for ETs avoidance. While a bunch of data is available on bacterial nucleases or other counter mechanisms, parasites have been neglected on this topic. Taken together, we call for more parasite-related studies in the exciting field of ETosis.

## Figures and Tables

**Table 1:** List of apicomplexan and euglenozoan protozoa capable of inducing ETosis, host species, professional phagocytes triggering ETs, and molecular mechanisms involved in this cell death process.

Parasites	Host species	Professional phagocytes	ETosis dependency	References
*Eimeria bovis*	Bovine	PMN	NOX NE MPO CD11b ERK 1/2 p38 MAPK SOCE	Muñoz-Caro et al. [23]

*Eimeria arloingi*	Caprine	PMN	NOX	Silva et al. [24]

*Eimeria ninakohlyakimovae*	Caprine	PMN	NOX	Pérez et al. (submitted manuscript)
		Monocytes	NOX	Pérez et al. (submitted manuscript)

*Toxoplasma gondii*	Mouse	PMN	ERK 1/2	Abi Abdallah et al. [25]
	Harbour seal	PMN	NOX NE MPO SOCE	Reichel et al. [26]

*Besnoitia besnoiti*	Bovine	PMN	NOX NE MPO	Muñoz-Caro et al. [27]
	Bovine	Monocytes	NOX MPO	Muñoz-Caro et al. [28]

*Neospora caninum*	Bovine	PMN	NOX NE MPO ERK 1/2 p38 MAPK SOCE P2Y2 PAD4	Villagra-Blanco et al. (submitted manuscript)

*Cryptosporidium parvum*	Bovine	PMN	NOX NE MPO ERK 1/2 p38 MAPK SOCE	Muñoz-Caro et al. [14]

*Leishmania *spp.	Human	PMN	NOX NE PAD4	Rochael et al. [29]

*Trypanosoma cruzi*	Human	PMN	NOX TLR2 TLR4	Sousa-Rocha et al. [30]

## References

[1] Segal A. W. (2005). How neutrophils kill microbes. *Annual Review of Immunology*.

[2] Faurschou M., Borregaard N. (2003). Neutrophil granules and secretory vesicles in inflammation. *Microbes and Infection*.

[3] Bainton D. F., Ullyot J. L., Farquhar M. G. (1971). The development of neutrophilic polymorphonuclear leukocytes in human bone marrow. *The Journal of Experimental Medicine*.

[4] Borregaard N., Cowland J. B. (1997). Granules of the human neutrophilic polymorphonuclear leukocyte. *Blood*.

[5] Nathan C. (2006). Neutrophils and immunity: challenges and opportunities. *Nature Reviews Immunology*.

[6] Hermosilla C., Caro T. M., Silva L. M. R., Ruiz A., Taubert A. (2014). The intriguing host innate immune response: Novel anti-parasitic defence by neutrophil extracellular traps. *Parasitology*.

[7] Underhill D. M., Ozinsky A. (2002). Phagocytosis of microbes: complexity in action. *Annual Review of Immunology*.

[8] Soehnlein O. (2009). Direct and alternative antimicrobial mechanisms of neutrophil-derived granule proteins. *Journal of Molecular Medicine*.

[9] Gullberg U., Andersson E., Garwicz D., Lindmark A., Olsson I. (1997). Biosynthesis, processing and sorting of neutrophil proteins: insight into neutrophil granule development. *European Journal of Haematology*.

[10] Borregaard N., Sørensen O. E., Theilgaard-Mönch K. (2007). Neutrophil granules: a library of innate immunity proteins. *Trends in Immunology*.

[11] Bliss S. K., Marshall A. J., Zhang Y., Denkers E. Y. (1999). Human polymorphonuclear leukocytes produce IL-12, TNF-*α*, and the chemokines macrophage-inflammatory protein-1*α* and -1*β* in response to *Toxoplasma gondii* antigens. *The Journal of Immunology*.

[12] Del Rio L., Butcher B. A., Bennouna S., Hieny S., Sher A., Denkers E. Y. (2004). *Toxoplasma gondii* triggers myeloid differentiation factor 88-dependent IL-12 and chemokine ligand 2 (monocyte chemoattractant protein 1) responses using distinct parasite molecules and host receptors. *The Journal of Immunology*.

[13] Behrendt J. H., Hermosilla C., Hardt M., Failing K., Zahner H., Taubert A. (2008). PMN-mediated immune reactions against *Eimeria bovis*. *Veterinary Parasitology*.

[14] Muñoz-Caro T., Lendner M., Daugschies A., Hermosilla C., Taubert A. (2015). NADPH oxidase, MPO, NE, ERK1/2, p38 MAPK and Ca^2+^ influx are essential for *Cryptosporidium parvum*-induced NET formation. *Developmental and Comparative Immunology*.

[15] Maksimov P., Hermosilla C., Kleinertz S., Hirzmann J., Taubert A. (2016). *Besnoitia besnoiti* infections activate primary bovine endothelial cells and promote PMN adhesion and NET formation under physiological flow condition. *Parasitology Research*.

[16] Kato T., Kitagawa S. (2006). Regulation of neutrophil functions by proinflammatory cytokines. *International Journal of Hematology*.

[17] Hachicha M., Rathanaswami P., Naccache P. H., McColl S. R. (1998). Regulation of chemokine gene expression in human peripheral blood neutrophils phagocytosing microbial pathogens. *Journal of Immunology*.

[18] Soehnlein O., Kenne E., Rotzius P., Eriksson E. E., Lindbom L. (2008). Neutrophil secretion products regulate anti-bacterial activity in monocytes and macrophages. *Clinical and Experimental Immunology*.

[19] Soehnlein O. (2009). An elegant defense: how neutrophils shape the immune response. *Trends in Immunology*.

[20] Borregaard N. (2010). Neutrophils, from marrow to microbes. *Immunity*.

[21] Cassatella M. A., Gasperini S., Calzetti F., Bertagnin A., Luster A. D., McDonald P. P. (1997). Regulated production of the interferon-*γ*-inducible protein-l0 (IP-10) chemokine by human neutrophils. *European Journal of Immunology*.

[22] Schorn C., Janko C., Latzko M., Chaurio R., Schett G., Herrmann M. (2012). Monosodium urate crystals induce extracellular DNA traps in neutrophils, eosinophils, and basophils but not in mononuclear cells. *Frontiers in Immunology*.

[23] Muñoz-Caro T., Mena Huertas S. J., Conejeros I. (2015). *Eimeria bovis*-triggered neutrophil extracellular trap formation is CD11b-, ERK 1/2-, p38 MAP kinase- and SOCE-dependent. *Veterinary Research*.

[24] Silva L. M. R., Muñoz Caro T., Gerstberger R. (2014). The apicomplexan parasite *Eimeria arloingi* induces caprine neutrophil extracellular traps. *Parasitology Research*.

[25] Abdallah D. S. Abi, Lin C., Ball C. J., King M. R., Duhamel G. E., Denkers E. Y. (2012). *Toxoplasma gondii* triggers release of human and mouse neutrophil extracellular traps. *Infection and Immunity*.

[26] Reichel M., Muñoz-Caro T., Sanchez Contreras G. (2015). Harbour seal (*Phoca vitulina*) PMN and monocytes release extracellular traps to capture the apicomplexan parasite *Toxoplasma gondii*. *Developmental and Comparative Immunology*.

[27] Muñoz-Caro T., Machado Ribeiro da Silva L., Rentería-Solis Z., Taubert A., Hermosilla C. (2016). Neutrophil extracellular traps in the intestinal mucosa of *Eimeria*-infected animals. *Asian Pacific Journal of Tropical Biomedicine*.

[28] Muñoz-Caro T., Silva L. M. R., Ritter C., Taubert A., Hermosilla C. (2014). * Besnoitia besnoiti* tachyzoites induce monocyte extracellular trap formation. *Parasitology Research*.

[29] Rochael N. C., Guimarães-Costa A. B., Nascimento M. T. C. (2015). Classical ROS-dependent and early/rapid ROS-independent release of Neutrophil extracellular traps triggered by *Leishmania* parasites. *Scientific Reports*.

[30] Sousa-Rocha D., Thomaz-Tobias M., Diniz L. F. A., Souza P. S. S., Pinge-Filho P., Toledo K. A. (2015). *Trypanosoma cruzi* and its soluble antigens induce NET release by stimulating toll-like receptors. *PLoS ONE*.

[31] Brinkmann V., Reichard U., Goosmann C. (2004). Neutrophil extracellular traps kill bacteria. *Science*.

[32] Lögters T., Margraf S., Altrichter J. (2009). The clinical value of neutrophil extracellular traps. *Medical Microbiology and Immunology*.

[33] Cheng O. Z., Palaniyar N. (2013). NET balancing: a problem in inflammatory lung diseases. *Frontiers in Immunology*.

[34] Hahn S., Giaglis S., Chowdury C. S., Hösli I., Hasler P. (2013). Modulation of neutrophil NETosis: interplay between infectious agents and underlying host physiology. *Seminars in Immunopathology*.

[35] Hahn S., Giaglis S., Hoesli I., Hasler P. (2012). Neutrophil NETs in reproduction: from infertility to preeclampsia and the possibility of fetal loss. *Frontiers in Immunology*.

[36] Rebordão M. R., Carneiro C., Alexandre-Pires G. (2014). Neutrophil extracellular traps formation by bacteria causing endometritis in the mare. *Journal of Reproductive Immunology*.

[37] Wartha F., Beiter K., Normark S., Henriques-Normark B. (2007). Neutrophil extracellular traps: casting the NET over pathogenesis. *Current Opinion in Microbiology*.

[38] Fuchs T. A., Abed U., Goosmann C. (2007). Novel cell death program leads to neutrophil extracellular traps. *Journal of Cell Biology*.

[39] Beiter K., Wartha F., Albiger B., Normark S., Zychlinsky A., Henriques-Normark B. (2006). An endonuclease allows *Streptococcus pneumoniae* to escape from neutrophil extracellular traps. *Current Biology*.

[40] Clark S. R., Ma A. C., Tavener S. A. (2007). Platelet TLR4 activates neutrophil extracellular traps to ensnare bacteria in septic blood. *Nature Medicine*.

[41] Aulik N. A., Hellenbrand K. M., Klos H., Czuprynski C. J. (2010). *Mannheimia haemolytica* and its leukotoxin cause neutrophil extracellular trap formation by bovine neutrophils. *Infection and Immunity*.

[42] Urban C. F., Reichard U., Brinkmann V., Zychlinsky A. (2006). Neutrophil extracellular traps capture and kill *Candida albicans* yeast and hyphal forms. *Cellular Microbiology*.

[43] Urban C. F., Ermert D., Schmid M. (2009). Neutrophil extracellular traps contain calprotectin, a cytosolic protein complex involved in host defense against *Candida albicans*. *PLoS Pathogens*.

[44] Byrd A. S., O'Brien X. M., Johnson C. M., Lavigne L. M., Reichner J. S. (2013). An extracellular matrix-based mechanism of rapid neutrophil extracellular trap formation in response to *Candida albicans*. *Journal of Immunology*.

[45] Ng T. H., Chang S.-H., Wu M.-H., Wang H.-C. (2013). Shrimp hemocytes release extracellular traps that kill bacteria. *Developmental and Comparative Immunology*.

[46] Narasaraju T., Yang E., Samy R. P. (2011). Excessive neutrophils and neutrophil extracellular traps contribute to acute lung injury of influenza pneumonitis. *American Journal of Pathology*.

[47] Jenne C. N., Kubes P. (2012). NETs tangle with HIV. *Cell Host and Microbe*.

[48] Jenne C. N., Kubes P. (2015). Virus-induced NETs—critical component of host defense or pathogenic mediator?. *PLoS Pathogens*.

[49] Saitoh T., Komano J., Saitoh Y. (2012). Neutrophil extracellular traps mediate a host defense response to human immunodeficiency virus-1. *Cell Host & Microbe*.

[50] Baker V. S., Imade G. E., Molta N. B. (2008). Cytokine-associated neutrophil extracellular traps and antinuclear antibodies in *Plasmodium falciparum* infected children under six years of age. *Malaria Journal*.

[51] Guimarães-Costa A. B., Nascimento M. T. C., Froment G. S. (2009). *Leishmania amazonensis* promastigotes induce and are killed by neutrophil extracellular traps. *Proceedings of the National Academy of Sciences of the United States of America*.

[52] Behrendt J. H., Ruiz A., Zahner H., Taubert A., Hermosilla C. (2010). Neutrophil extracellular trap formation as innate immune reactions against the apicomplexan parasite *Eimeria bovis*. *Veterinary Immunology and Immunopathology*.

[53] Bonne-Année S., Kerepesi L. A., Hess J. A. (2014). Extracellular traps are associated with human and mouse neutrophil and macrophage mediated killing of larval *Strongyloides stercoralis*. *Microbes and Infection*.

[54] Branzk N., Lubojemska A., Hardison S. E. (2014). Neutrophils sense microbe size and selectively release neutrophil extracellular traps in response to large pathogens. *Nature Immunology*.

[55] Muñoz-Caro T., Rubio R M. C., Silva L. M. R. (2015). Leucocyte-derived extracellular trap formation significantly contributes to *Haemonchus contortus* larval entrapment. *Parasites and Vectors*.

[56] Yousefi S., Mihalache C., Kozlowski E., Schmid I., Simon H. U. (2009). Viable neutrophils release mitochondrial DNA to form neutrophil extracellular traps. *Cell Death and Differentiation*.

[57] Martinelli S., Urosevic M., Baryadel A. (2004). Induction of genes mediating interferon-dependent extracellular trap formation during neutrophil differentiation. *Journal of Biological Chemistry*.

[58] Caudrillier A., Kessenbrock K., Gilliss B. M. (2012). Platelets induce neutrophil extracellular traps in transfusion-related acute lung injury. *The Journal of Clinical Investigation*.

[59] Muñoz Caro T., Hermosilla C., Silva L. M. R., Cortes H., Taubert A. (2014). Neutrophil extracellular traps as innate immune reaction against the emerging apicomplexan parasite *Besnoitia besnoiti*. *PloS ONE*.

[60] Nishinaka Y., Arai T., Adachi S., Takaori-Kondo A., Yamashita K. (2011). Singlet oxygen is essential for neutrophil extracellular trap formation. *Biochemical and Biophysical Research Communications*.

[61] Pijanowski L., Scheer M., Verburg-van Kemenade B. M. L., Chadzinska M. (2015). Production of inflammatory mediators and extracellular traps by carp macrophages and neutrophils in response to lipopolysaccharide and/or interferon-*γ*2. *Fish and Shellfish Immunology*.

[62] Gupta A. K., Hasler P., Holzgreve W., Gebhardt S., Hahn S. (2005). Induction of neutrophil extracellular DNA lattices by placental microparticles and IL-8 and their presence in preeclampsia. *Human Immunology*.

[63] Neeli I., Khan S. N., Radic M. (2008). Histone deimination as a response to inflammatory stimuli in neutrophils. *The Journal of Immunology*.

[64] Wang Y., Li M., Stadler S. (2009). Histone hypercitrullination mediates chromatin decondensation and neutrophil extracellular trap formation. *The Journal of Cell Biology*.

[65] Papayannopoulos V., Metzler K. D., Hakkim A., Zychlinsky A. (2010). Neutrophil elastase and myeloperoxidase regulate the formation of neutrophil extracellular traps. *The Journal of Cell Biology*.

[66] Hakkim A., Fuchs T. A., Martinez N. E. (2011). Activation of the Raf-MEK-ERK pathway is required for neutrophil extracellular trap formation. *Nature Chemical Biology*.

[67] Douda D. N., Khan M. A., Grasemann H., Palaniyar N. (2015). SK3 channel and mitochondrial ROS mediate NADPH oxidase-independent NETosis induced by calcium influx. *Proceedings of the National Academy of Sciences of the United States of America*.

[68] Pilsczek F. H., Salina D., Poon K. K. H. (2010). A novel mechanism of rapid nuclear neutrophil extracellular trap formation in response to *Staphylococcus aureus*. *The Journal of Immunology*.

[69] Keshari R. S., Jyoti A., Dubey M. (2012). Cytokines induced neutrophil extracellular traps formation: implication for the inflammatory disease condition. *PLoS ONE*.

[70] Palić D., Ostojić J., Andreasen C. B., Roth J. A. (2007). Fish cast NETs: neutrophil extracellular traps are released from fish neutrophils. *Developmental and Comparative Immunology*.

[71] Burgos R. A., Conejeros I., Hidalgo M. A., Werling D., Hermosilla C. (2011). Calcium influx, a new potential therapeutic target in the control of neutrophil-dependent inflammatory diseases in bovines. *Veterinary Immunology and Immunopathology*.

[72] Gupta A. K., Giaglis S., Hasler P., Hahn S. (2014). Efficient neutrophil extracellular trap induction requires mobilization of both intracellular and extracellular calcium pools and is modulated by cyclosporine A. *PLoS ONE*.

[73] Li P., Li M., Lindberg M. R., Kennett M. J., Xiong N., Wang Y. (2010). PAD4 is essential for antibacterial innate immunity mediated by neutrophil extracellular traps. *Journal of Experimental Medicine*.

[74] Leshner M., Wang S., Lewis C. (2012). PAD4 mediated histone hypercitrullination induces heterochromatin decondensation and chromatin unfolding to form neutrophil extracellular trap-like structures. *Frontiers in Immunology*.

[75] Brinkmann V., Zychlinsky A. (2012). Neutrophil extracellular traps: is immunity the second function of chromatin?. *The Journal of Cell Biology*.

[76] Von Köckritz-Blickwede M., Nizet V. (2009). Innate immunity turned inside-out: antimicrobial defense by phagocyte extracellular traps. *Journal of Molecular Medicine*.

[77] Arai Y., Nishinaka Y., Arai T. (2014). Uric acid induces NADPH oxidase-independent neutrophil extracellular trap formation. *Biochemical and Biophysical Research Communications*.

[78] Grinberg N., Elazar S., Rosenshine I., Shpigel N. Y. (2008). *β*-Hydroxybutyrate abrogates formation of bovine neutrophil extracellular traps and bactericidal activity against mammary pathogenic *Escherichia coli*. *Infection and Immunity*.

[79] Ramos-Kichik V., Mondragón-Flores R., Mondragón-Castelán M. (2009). Neutrophil extracellular traps are induced by *Mycobacterium tuberculosis*. *Tuberculosis*.

[80] Aulik N. A., Hellenbrand K. M., Czuprynski C. J. (2012). *Mannheimia haemolytica* and its leukotoxin cause macrophage extracellular trap formation by bovine macrophages. *Infection and Immunity*.

[81] Hellenbrand K. M., Forsythe K. M., Rivera-Rivas J. J., Czuprynski C. J., Aulik N. A. (2013). *Histophilus somni* causes extracellular trap formation by bovine neutrophils and macrophages. *Microbial Pathogenesis*.

[82] Boe D. M., Curtis B. J., Chen M. M., Ippolito J. A., Kovacs E. J. (2015). Extracellular traps and macrophages: new roles for the versatile phagocyte. *Journal of Leukocyte Biology*.

[83] Chow O. A., von Köckritz-Blickwede M., Bright A. T. (2010). Statins enhance formation of phagocyte extracellular traps. *Cell Host and Microbe*.

[84] Von Köckritz-Blickwede M., Goldmann O., Thulin P. (2008). Phagocytosis-independent antimicrobial activity of mast cells by means of extracellular trap formation. *Blood*.

[85] Lin A. M., Rubin C. J., Khandpur R. (2011). Mast cells and neutrophils release IL-17 through extracellular trap formation in psoriasis. *Journal of Immunology*.

[86] Yousefi S., Gold J. A., Andina N. (2008). Catapult-like release of mitochondrial DNA by eosinophils contributes to antibacterial defense. *Nature Medicine*.

[87] Dworski R., Simon H.-U., Hoskins A., Yousefi S. (2011). Eosinophil and neutrophil extracellular DNA traps in human allergic asthmatic airways. *Journal of Allergy and Clinical Immunology*.

[88] Morshed M., Hlushchuk R., Simon D. (2014). NADPH oxidase-independent formation of extracellular DNA traps by basophils. *Journal of Immunology*.

[89] Malawista S. E., Van Blaricom G., Breitenstein M. G. (1989). Cryopreservable neutrophil surrogates. Stored cytoplasts from human polymorphonuclear leukocytes retain chemotactic, phagocytic, and microbicidal function. *Journal of Clinical Investigation*.

[90] Yipp B. G., Petri B., Salina D. (2012). Infection-induced NETosis is a dynamic process involving neutrophil multitasking in vivo. *Nature Medicine*.

[91] Schauer C., Janko C., Munoz L. E. (2014). Aggregated neutrophil extracellular traps limit inflammation by degrading cytokines and chemokines. *Nature Medicine*.

[92] WHO (2014). *World Malaria Report 2014*.

[93] Waisberg M., Molina-Cruz A., Mizurini D. M. (2014). *Plasmodium falciparum* infection induces expression of a mosquito salivary protein (Agaphelin) that targets neutrophil function and inhibits thrombosis without impairing hemostasis. *PLoS Pathogens*.

[94] Chapman H. D. (2014). Milestones in avian coccidiosis research: a review. *Poultry Science*.

[95] Hermosilla C., Barbisch B., Heise A., Kowalik S., Zahner H. (2002). Development of *Eimeria boris* in vitro: suitability of several bovine, human and porcine endothelial cell lines, bovine fetal gastrointestinal, Madin-Darby bovine kidney (MDBK) and African green monkey kidney (VERO) cells. *Parasitology Research*.

[96] Hermosilla C., Bürger H.-J., Zahner H. (1999). T cell responses in calves to a primary *Eimeria bovis* infection: phenotypical and functional changes. *Veterinary Parasitology*.

[97] Daugschies A., Najdrowski M. (2005). Eimeriosis in cattle: current understanding. *Journal of Veterinary Medicine Series B: Infectious Diseases and Veterinary Public Health*.

[98] Faber J.-E., Kollmann D., Heise A. (2002). *Eimeria* infections in cows in the periparturient phase and their calves: oocyst excretion and levels of specific serum and colostrum antibodies. *Veterinary Parasitology*.

[99] Ruiz A., González J. F., Rodríguez E. (2006). Influence of climatic and management factors on *Eimeria* infections in goats from semi-arid zones. *Journal of Veterinary Medicine Series B: Infectious Diseases and Veterinary Public Health*.

[100] Soe A. K., Pomroy W. E. (1992). New species of *Eimeria* (Apicomplexa: Eimeriidae) from the domesticated goat *Capra hircus* in New Zealand. *Systematic Parasitology*.

[101] Mehlhorn H., Armstrong P. M. (2001). *Encyclopedic Reference of Parasitology: Biology, Structure, Function*.

[102] Silva L. M., Vila-Viçosa M. J., Nunes T., Taubert A., Hermosilla C., Cortes H. C. (2014). Eimeria infections in goats in Southern Portugal. *Revista Brasileira de Parasitologia Veterinária*.

[103] Taylor M. A., Catchpole J. (1994). Review article: coccidiosis of domestic ruminants. *Applied Parasitology*.

[104] Friend S. C. E., Stockdale P. H. G. (1980). Experimental *Eimeria bovis* infection in calves: a histopathological study. *Canadian Journal of Comparative Medicine*.

[105] Ruiz A., Matos L., Muñoz M. C. (2013). Isolation of an *Eimeria ninakohlyakimovae* field strain (Canary Islands) and analysis of its infection characteristics in goat kids. *Research in Veterinary Science*.

[106] Mesfin G. M., Bellamy J. E. C., Stockdale P. H. G. (1978). The pathological changes caused by *Eimeria falciformis* var pragensis in mice. *Canadian Journal of Comparative Medicine*.

[107] Gadde U., Chapman H. D., Rathinam T., Erf G. F. (2011). Cellular immune responses, chemokine, and cytokine profiles in Turkey poults following infection with the intestinal parasite *Eimeria adenoeides*. *Poultry Science*.

[108] Szabady R. L., McCormick B. A. (2013). Control of neutrophil inflammation at mucosal surfaces by secreted epithelial products. *Frontiers in Immunology*.

[109] Sumagin R., Robin A. Z., Nusrat A., Parkos C. A. (2014). Transmigrated neutrophils in the intestinal lumen engage ICAM-1 to regulate the epithelial barrier and neutrophil recruitment. *Mucosal Immunology*.

[110] Hosseinzadeh A., Messer P. K., Urban C. F. (2012). Stable redox-cycling nitroxidetempol inhibits NET formation. *Frontiers in Immunology*.

[111] Taubert A., Behrendt J. H., Sühwold A., Zahner H., Hermosilla C. (2009). Monocyte- and macrophage-mediated immune reactions against *Eimeria bovis*. *Veterinary Parasitology*.

[112] Chuah C., Jones M. K., Burke M. L., Mcmanus D. P., Owen H. C., Gobert G. N. (2014). Defining a pro-inflammatory neutrophil phenotype in response to schistosome eggs. *Cellular Microbiology*.

[113] Chuah C., Jones M. K., Burke M. L. (2013). Spatial and temporal transcriptomics of *Schistosoma japonicum*-induced hepatic granuloma formation reveals novel roles for neutrophils. *Journal of Leukocyte Biology*.

[114] Hill D., Dubey J. P. (2002). *Toxoplasma gondii*: transmission, diagnosis, and prevention. *Clinical Microbiology and Infection*.

[115] Dubey J. P. (2009). History of the discovery of the life cycle of *Toxoplasma gondii*. *International Journal for Parasitology*.

[116] Tenter A. M., Heckeroth A. R., Weiss L. M. (2000). *Toxoplasma gondii*: from animals to humans. *International Journal for Parasitology*.

[117] Bliss S. K., Butcher B. A., Denkers E. Y. (2000). Rapid recruitment of neutrophils containing prestored IL-12 during microbial infection. *Journal of Immunology*.

[118] MacLaren A., Attias M., De Souza W. (2004). Aspects of the early moments of interaction between tachyzoites of *Toxoplasma gondii* with neutrophils. *Veterinary Parasitology*.

[119] MacLaren A., De Souza W. (2002). Further studies on the interaction of *Toxoplasma gondii* with neutrophils and eosinophils. *Journal of Submicroscopic Cytology and Pathology*.

[120] Abi Abdallah D. S., Denkers E. Y. (2012). Neutrophils cast extracellular traps in response to protozoan parasites. *Frontiers in Immunology*.

[121] Harker K. S., Ueno N., Wang T., Bonhomme C., Liu W., Lodoen M. B. (2013). *Toxoplasma gondii* modulates the dynamics of human monocyte adhesion to vascular endothelium under fluidic shear stress. *Journal of Leukocyte Biology*.

[122] Ueno N., Harker K. S., Clarke E. V. (2014). Real-time imaging of *Toxoplasma*-infected human monocytes under fluidic shear stress reveals rapid translocation of intracellular parasites across endothelial barriers. *Cellular Microbiology*.

[123] Quan J.-H., Zhou W., Cha G.-H., Choi I.-W., Shin D.-W., Lee Y.-H. (2013). Kinetics of IL-23 and IL-12 secretion in response to *Toxoplasma gondii* antigens from THP-1 monocytic cells. *The Korean Journal of Parasitology*.

[124] Dubey J. P., van Wilpe E., Blignaut D. J. C., Schares G., Williams J. H. (2013). Development of early tissue cysts and associated pathology of *Besnoitia besnoiti* in a naturally infected bull (*Bos taurus*) from South Africa. *Journal of Parasitology*.

[125] Schares G., Basso W., Majzoub M. (2011). Evaluation of a commercial ELISA for the specific detection of antibodies against *Besnoitia besnoiti*. *Veterinary Parasitology*.

[126] Schares G., Langenmayer M. C., Scharr J. C. (2013). Novel tools for the diagnosis and differentiation of acute and chronic bovine besnoitiosis. *International Journal for Parasitology*.

[127] EFSA (2010). Bovine besnoitiosis: an emerging disease in Europe. *EFSA Journal*.

[128] Álvarez-García G., Frey C. F., Mora L. M. O., Schares G. (2013). A century of bovine besnoitiosis: an unknown disease re-emerging in Europe. *Trends in Parasitology*.

[129] Gupta A. K., Joshi M. B., Philippova M. (2010). Activated endothelial cells induce neutrophil extracellular traps and are susceptible to NETosis-mediated cell death. *FEBS Letters*.

[130] Saffarzadeh M., Juenemann C., Queisser M. A. (2012). Neutrophil extracellular traps directly induce epithelial and endothelial cell death: a predominant role of histones. *PLoS ONE*.

[131] Carmona-Rivera C., Zhao W., Yalavarthi S., Kaplan M. J. (2015). Neutrophil extracellular traps induce endothelial dysfunction in systemic lupus erythematosus through the activation of matrix metalloproteinase-2. *Annals of the Rheumatic Diseases*.

[132] Etulain J., Martinod K., Wong S. L., Cifuni S. M., Schattner M., Wagner D. D. (2015). P-selectin promotes neutrophil extracellular trap formation in mice. *Blood*.

[133] Lendner M., Daugschies A. (2014). *Cryptosporidium* infections: molecular advances. *Parasitology*.

[134] Shahiduzzaman M., Daugschies A. (2012). Therapy and prevention of cryptosporidiosis in animals. *Veterinary Parasitology*.

[135] Kourenti C., Karanis P., Smith H. (2007). Waterborne transmission of protozoan parasites: a worldwide review of outbreaks and lessons learnt. *Journal of Water and Health*.

[136] Takeuchi D., Jones V. C., Kobayashi M., Suzuki F. (2008). Cooperative role of macrophages and neutrophils in host antiprotozoan resistance in mice acutely infected with *Cryptosporidium parvum*. *Infection and Immunity*.

[137] Lacroix-Lamandé S., Mancassola R., Naciri M., Laurent F. (2002). Role of gamma interferon in chemokine expression in the ileum of mice and in a murine intestinal epithelial cell line after *Cryptosporidium parvum* infection. *Infection and Immunity*.

[138] Brazil J. C., Liu R., Sumagin R. (2013). *α*3/4 fucosyltransferase 3-dependent synthesis of sialyl lewis A on CD44 variant containing exon 6 mediates polymorphonuclear leukocyte detachment from intestinal epithelium during transepithelial migration. *Journal of Immunology*.

[139] Seper A., Hosseinzadeh A., Gorkiewicz G. (2013). *Vibrio cholerae* evades neutrophil extracellular traps by the activity of two extracellular nucleases. *PLoS Pathogens*.

[140] Alfaro C., Teijeira A., Onate C. (2016). Tumor-produced interleukin-8 attracts human myeloid-derived suppressor cells and elicits extrusion of neutrophil extracellular traps (NETs). *Clinical Cancer Research*.

[141] Allam R., Kumar S. V. R., Darisipudi M. N., Anders H.-J. (2014). Extracellular histones in tissue injury and inflammation. *Journal of Molecular Medicine*.

[142] Ashford R. W. (2000). The leishmaniases as emerging and reemerging zoonoses. *International Journal for Parasitology*.

[143] Alvar J., Vélez I. D., Bern C. (2012). Leishmaniasis worldwide and global estimates of its incidence. *PLoS ONE*.

[144] Kolivand M., Fallah M., Salehzadeh A. (2015). An epidemiological study of cutaneous leishmaniasis using active case finding among elementary school students in Pakdasht, Southeast of Tehran, Iran 2013-2014. *Journal of Research in Health Sciences*.

[145] Chagas A. C., Oliveira F., Debrabant A., Valenzuela J. G., Ribeiro J. M. C., Calvo E. (2014). Lundep, a sand fly salivary endonuclease increases *Leishmania* parasite survival in neutrophils and inhibits xiia contact activation in human plasma. *PLoS Pathogens*.

[146] Charmoy M., Auderset F., Allenbach C., Tacchini-Cottier F. (2010). The prominent role of neutrophils during the initial phase of infection by *Leishmania* parasites. *Journal of Biomedicine and Biotechnology*.

[147] Hurrell B. P., Schuster S., Grün E. (2015). Rapid sequestration of *Leishmania mexicana* by Neutrophils contributes to the development of chronic lesion. *PLoS Pathogens*.

[148] Peters N. C., Egen J. G., Secundino N. (2008). In vivo imaging reveals an essential role for neutrophils in leishmaniasis transmitted by sand flies. *Science*.

[149] Thalhofer C. J., Chen Y., Sudan B., Love-Homan L., Wilson M. E. (2011). Leukocytes infiltrate the skin and draining lymph nodes in response to the protozoan *Leishmania infantum chagasi*. *Infection and Immunity*.

[150] Gabriel C., McMaster W. R., Girard D., Descoteaux A. (2010). *Leishmania donovani* promastigotes evade the antimicrobial activity of neutrophil extracellular traps. *Journal of Immunology*.

[151] Morgado F. N., Nascimento M. T. C., Saraiva E. M. (2015). Are neutrophil extracellular traps playing a role in the parasite control in active American tegumentary leishmaniasis lesions?. *PLoS ONE*.

[152] Guimarães-Costa A. B., DeSouza-Vieira T. S., Paletta-Silva R., Freitas-Mesquita A. L., Meyer-Fernandes J. R., Saraiva E. M. (2014). 3′-nucleotidase/nuclease activity allows *Leishmania* parasites to escape killing by neutrophil extracellular traps. *Infection and Immunity*.

[153] Wang Y., Chen Y., Xin L. (2011). Differential microbicidal effects of human histone proteins H2A and H2B on *Leishmania* promastigotes and amastigotes. *Infection and Immunity*.

[154] Chagas disease. http://www.paho.org/hq/index.php?option=com_topics&view=article&id=10&Itemid=40743.

[155] Villalta F., Kierszenbaum F. (1984). Host-cell invasion by *Trypanosoma cruzi*: role of cell surface galactose residues. *Biochemical and Biophysical Research Communications*.

[156] Luna-Gomes T., Filardy A. A., Rocha J. D. B. (2014). Neutrophils increase or reduce parasite burden in *Trypanosoma cruzi*-infected macrophages, depending on host strain: role of neutrophil elastase. *PLoS ONE*.

